# The Combination of Amoxicillin-Clavulanic Acid and Ketoconazole in the Treatment of *Madurella mycetomatis* Eumycetoma and *Staphylococcus aureus* Co-infection

**DOI:** 10.1371/journal.pntd.0002959

**Published:** 2014-06-19

**Authors:** Najwa A. Mhmoud, Ahmed Hassan Fahal, El Sheikh Mahgoub, Wendy W. J. van de Sande

**Affiliations:** 1 Mycetoma Research Centre, University of Khartoum, Khartoum, Sudan; 2 Department of Medical Microbiology and Infectious Diseases, Erasmus Medical Centre Rotterdam, The Netherlands; University of Tennessee, United States of America

## Abstract

Eumycetoma is a chronic progressive disabling and destructive inflammatory disease which is commonly caused by the fungus *Madurella mycetomatis*. It is characterized by the formation of multiple discharging sinuses. It is usually treated by antifungal agents but it is assumed that the therapeutic efficiency of these agents is reduced by the co-existence of *Staphylococcus aureus* co-infection developing in these sinuses. This prospective study was conducted to investigate the safety, efficacy and clinical outcome of combined antibiotic and antifungal therapy in eumycetoma patients with superimposed *Staphylococcus aureus* infection. The study enrolled 337 patients with confirmed *M. mycetomatis* eumycetoma and *S. aureus* co-infection. Patients were allocated into three groups; 142 patients received amoxicillin-clavulanic acid and ketoconazole, 93 patients received ciprofloxacin and ketoconazole and 102 patients received ketoconazole only. The study showed that, patients who received amoxicillin-clavulanic acid and ketoconazole treatment had an overall better clinical outcome compared to those who had combined ciprofloxacin and ketoconazole or to those who received ketoconazole only. In this study, 60.6% of the combined amoxicillin-clavulanic acid/ketoconazole group showed complete or partial clinical response to treatment compared to 30.1% in the ciprofloxacin/ketoconazole group and 36.3% in the ketoconazole only group. The study also showed that 64.5% of the patients in the ciprofloxacin/ketoconazole group and 59.8% in the ketoconazole only group had progressive disease and poor outcome. This study showed that the combination of amoxicillin-clavulanic acid and ketoconazole treatment is safe and offers good clinical outcome and it is therefore recommended to treat eumycetoma patients with *Staphylococcus aureus* co-infection.

## Author Summary

Mycetoma, a unique neglected tropical disease, is characterised by massive deformity and disability presenting with painless subcutaneous mass, multiple sinuses that produce sero-purulent discharge. Secondary bacterial infection is common in mycetoma and it is assumed that, this bacterial co-infection reduces the effectiveness of the antifungal treatment and is responsible for the poor treatment outcome. To investigate this hypothesis, this study examined the combination of Ketoconazole and antibiotics in two groups of patients with eumycetoma and *Staphylococcus aureus* (*S. aureus*) co-infection. The first group received ketoconazole and amoxicillin-clavulanic and the second group received ciprofloxacin and ketoconazole. The study also included a historical group of patients who had only ketoconazole as a control group. Patients who had amoxicillin-clavulanic acid and ketoconazole had an overall better clinical outcome and superior infection eradication compared to the two other groups. It can be concluded that the combination of amoxicillin-clavulanic acid and ketoconazole is safe treatment and offers better clinical outcome in eumycetoma patients with *S. aureus* co-infection.

## Introduction

Mycetoma is a chronic granulomatous subcutaneous infection which is endemic in the tropical and subtropical regions of Central and South-America, Asia and Africa. It is caused by either bacteria (actinomycetoma) or fungi (eumycetoma). Eumycetoma is highly endemic in Sudan and commonly caused by the fungus *Madurella mycetomatis* [1], [2], [3], [4]. Clinically, mycetoma presents as a slowly progressive painless subcutaneous swelling commonly at the site of previous trauma. Multiple secondary nodules evolve within the swelling, suppurate and drain serous, sero-sanguinous or purulent discharge [1]–[3]. The discharge is initially sterile, but later on, may become exposed to secondary bacterial infections due to poor hygiene [5]. Routine monitoring follow-up visits of these patients at the Mycetoma Research Centre (MRC) of the University of Khartoum, Sudan documented recurrent bacterial co-infections with *S. aureus* infection [3], [5].

Eumycetoma continues to pose management challenges. The current management approach consists of a prolonged course of drugs combined with surgery. Unfortunately, the cure rate is rather low with a high recurrence rate [6]-[8]. Therefore there is a desperate need to make available novel antifungal agents and to develop new approaches in the endemic areas.

Several studies have confirmed that, the majority of the *S. aureus* infection is of endogenous origin and nasal carriage of *S. aureus* is the risk factor for developing a co-infection in many skin and soft tissue infections [9]–[18]. In general, local bacterial co-infections may disturb the local tissue environment reducing the antifungal drug efficacy, producing local metabolites which are likely to induce changes that affect the healing process and increase drug resistance [19], [20]. Therefore controlling bacterial co-infection by the use of appropriate antibiotics could normalize the tissue environment and therefore augment the efficacy of antifungal agents in the management of eumycetoma.

With this background, this prospective study was set out to determine the safety and efficacy of the combination of ketoconazole and amoxicillin-clavulanic acid or ciprofloxacin as a first line treatment for patients with eumycetoma caused by *M. mycetomatis* and *S. aureus* co-infection.

## Materials and Methods

### Study Design

This prospective study was conducted at the Mycetoma Research Centre, University of Khartoum in the period between January 2011 and June 2013. It included 337 consecutive patients with confirmed *Madurella mycetomatis* eumycetoma; based on a confirmed grains culture, fine needle aspiration for cytology and/or histopathologic examination of surgical biopsies. All patients had a *S. aureus* co-infection at the time of enrollment. None of the patients had previous surgical or medical treatment for eumycetoma.

All patients were carefully interviewed. Demographic information was gathered on gender, age, social habits, co-morbidities and history of hospitalization in the past year. All patients underwent a thorough general and local physical examination. This included lesion size in cm from fixed points, the number of active sinuses, skin attachment to deep structures and disability.

The study population was divided according to their lesions' size into three groups; massive lesion (>10 cm in diameter), moderate lesion (5–10 cm in diameter) and small lesion (<5 cm in diameter).

All patients had full blood count examination, hepatic and renal function tests, radiological examination of the affected site (anterio-posterior and lateral views) and lesion ultrasound examination. Anterior nares and sinuses swab cultures for *S. aureus* were obtained from all patients as previously described [13], [14]. The bacteria were cultured and identified to the species level by Gram's staining, colonial morphology, biochemical testing; catalase, DNAse and Coagulase tests. A co-infection was defined as a deep wound infection as documented clinically by treating clinician and culture isolates.

According to the results obtained from the sinus swab culture, antibiotics were given in prolonged courses, each course lasted 12 weeks. Several courses were administered. In this study, the two common antibiotics that showed organism's sensitivity were amoxicillin-clavulanic acid and ciprofloxacin. Patients were allocated according to their bacteriology culture results into two groups; the amoxicillin-clavulanic acid and ketoconazole group and the ciprofloxacin and ketoconazole group. Ketoconazole was the eumycetoma drug of choice at the MRC during the study duration.

One hundred forty two patients received one gram amoxicillin-clavulanic acid orally twice daily combined with oral ketoconazole 400 mg twice daily and 93 patients received oral ciprofloxacin 500 mg twice daily combined with oral ketoconazole 400 mg twice daily. Another 102 patients from the MRC records who only received 400 mg ketoconazole orally twice daily were selected and included as a control group.

All patients were followed up closely every four weeks for clinical, hepatic and renal functions assessment. The medical treatment duration ranged between 6 months and one year depending on clinical response followed by wide surgical excision.

Cure and amputation were considered as study outcomes. Cure was defined as complete mass disappearance, healed sinuses, normal skin and absence of hyper-reflective echoes and cavities on ultra-sound examination [21].

### Ethical Clearance

Ethical clearance was obtained from Soba University Hospital Ethical Committee. Patients gave written informed consent.

### Statistical Analysis

One way ANOVA was used to compare the mean of age, duration and occupation for the three study groups. The clinical response to the combinations of ketoconazole and the antibiotics was assessed with the Fisher exact test. The significance of differences in patients and control were calculated and *p*-value of <0.05 considered statistically significant. All statistical analyses were performed using SPSS for Windows v11.0 statistical analysis software.

## Results

### Study Population

To test the hypothesis that eradicating secondary bacterial infections would have a positive effect on the outcome of eumycetoma treatment, 337 patients with confirmed *M. mycetomatis* and *S. aureus* co-infection were enrolled in this study (Table 1).

**Table 1:** Study population demographic features.

Characteristic		Amoxicillin-Clavulanic Acid Group	Ciprofloxacin Group	Ketoconazole Only Group	P-Value
**Number**		142	93	102)	
**Mean Age (Range)**		29.99 (7–.76)	27.78 (12–60)	24.86(9–57	0.262
**Gender (Male/Female)**		112/30	79/14	67/35	**0.002**
**Occupation**	Farmer	29(20.6)	24(25.8)	16(15.7)	0.058
	Workers	44(31.2)	26(28)	17(16.7)	
	Student	41(29.1)	24(25.8)	34(33.3)	
	Housewife	17(12.1)	14(15.1)	23(22.5)	
	Jobless	8(5.7)	5(5.4)	10(9.8)	
	Other Job	3(2.11)	0(0.0)	2(2.0)	
Mean Duration before treatment± SD (range)		7.62±7.094 (1–38 Year)	6.65±5.237 (1–23 Year)	6.32±5.136 (1–23 Year)	1.000

The *p* value<0.05 was deemed statistically significant. Significant *p* values are highlighted in the boldfaced letters.

All *S. aureus* strains were assessed for their susceptibility to a large panel of antimicrobial agents. All strains were susceptible to amoxicillin-Clavulanic acid and/or ciprofloxacin.

### Adding Amoxicillin-Clavulanic Acid but Not Ciprofloxacin to Ketoconazole Treatment Improves the Therapeutic Outcome of Ketoconazole Treated Mycetoma Patients with *S. aureus* Co-infection

The treatment duration in the amoxicillin-clavulanic acid group ranged between 3 and 12 months (mean 9.6 months) and the bacterial co-infection was eradicated in 97.2% of the patients. In the ciprofloxacin group, the treatment duration ranged between 3 and 12 months (mean 10 months) and in this group, none of the bacterial co-infection was eradicated.

Patients who had received a combination of amoxicillin-clavulanic acid and ketoconazole had an overall better clinical outcome than those received a combination of ciprofloxacin and ketoconazole or ketoconazole alone.

In the amoxicillin-clavulanic acid group, 39 patients (27.5%), showed complete and 47 patients (33.1%) had partial clinical response to treatment compared to 13 patients (14%) complete and 15 patients (16.1%) had partial response in the ciprofloxacin group. In the ketoconazole group 22 patients (21%) had complete response and 15 patients (14.7%) had partial response. Table 2,3.

**Table 2:** The mycetoma treatment outcome in patients with a secondary *S. aureus* infection when treated with amoxicillin clavulanic acid.

Clinical Evaluation	Amoxicillin-Clavulanic Acid Group (N = 142)	Ketoconazole Only (N = 102)	P-Value*
**Complete response**	**39** (27.5%)	**22 (21.6%)**	**0.000**
All sinuses closed and/or	36	03	
Lesion disappeared completely- without relapse	03	19	
**Partial Response:.**	**47 (33.1%)**	**15 (14.7%)**	0.547
>50% of sinuses are healed and/or	33	11	
>50% decrease of the swelling size	14	04	
**Stable Disease**	**42 (29.6%)**	**4 (3.9%)**	0.465
No change in sinuses and/or	23	03	
No change in lesion size	19	01	
**Progressive Disease**	**14 (9.9%)**	**61 (59.8%)**	0.130
Increased sinuses in number and/or	03	22	
Increased lesion size	11	39	

P-value as calculated by fisher exact test.

**Table 3:** The mycetoma treatment outcome in patients with a secondary *S. aureus* infection when treated with ciprofloxacin.

Clinical Evaluation	Ciprofloxacin Group (N = 93)	Ketoconazole Only (N = 102)	P-Value
**Complete response**	**13 (14.0%)**	**22(21.%)**	0.474
All sinuses closed and/or	03	03	
Lesion disappeared completely- without relapse	10	19	
**Partial Response:**	**15 (16.1%)**	**15 (14.7%)**	1.000
>50% of sinuses are healed and/or	11	11	
>50% decrease of the swelling size.	04	04	
**Stable Disease**	**5 (5.3%)**	**4 (3.9%)**	0.624
No change in sinuses and/or	01	03	
No change in lesion size	04	01	
**Progressive Disease**	**60 (64.5%)**	**61 (59.8%)**	0.704
Increased sinuses in number and/or	21	22	
Increased lesion size	39	39	

P-value as calculated by fisher exact test.

The study also showed that 60 patients (64.5%) of the ciprofloxacin group and 61 patients (59.8%) of the ketoconazole monotherapy group had progressive disease and poor outcome, (Table 3).

Based on these results, it can be concluded that, the combination of amoxicillin-Clavulanic acid and ketoconazole has superior outcome in the treatment of eumycetoma patients with *S. aureus* co-infection. This also suggests that treating co-infection influences the outcome of the eumycetoma treatment with ketoconazole.

### Adding Amoxicillin-Clavulanic Acid but Not Ciprofloxacin to Ketoconazole Treatment Improves the Skin Abnormalities, Pain and Mobility of Eumycetoma Patients with *S. aureus* Co-infection

In addition, to influencing the outcome of the ketoconazole therapy, we investigated if treating the co-infection also play a role in improving the skin abnormalities, the pain and even the disability observed in the eumycetoma patients.

At the end of the study, 34.5% of the amoxicillin-clavulanic acid patients group had skin appearance improvement in contra-distinction to patients of the other two groups. (Fisher exact, *p* = 0.000).

Although mycetoma is generally regarded as painless, pain can be induced by secondary bacterial infections. In the amoxicillin-clavulanic acid treated group significantly fewer patients (18 patients; 12.7%) suffered from pain in the lesion compared to the ciprofloxacin treated group (79 patients; 85%), (Fisher exact, *p* = 0.000).

At the end of the treatment period, the overall mobility of the patients who received amoxicillin-clavulanic acid was significantly better than those receiving ciprofloxacin. In the amoxicillin-clavulanic acid group 123 out of 139 patients, had no disability, versus 2 of the 93 in the ciprofloxacin treated group (Fisher exact, *p* = 0.000). The degree of disability was more severe in the ciprofloxacin treated group than in the amoxicillin-clavulanic acid treated group. Of the 91 patients with disabilities in the ciprofloxacin-treated group, 77 had severe disability, while only four out of the 16 in the amoxicillin-clavulanic acid treated group were severely disabled (Fisher exact, *p* = 0.00). Four patients in amoxicillin-clavulanic acid group and 11 patients in ciprofloxacin group had amputation.

### Combination of Amoxicillin-Clavulanic Acid or Ciprofloxacin and Ketoconazole for the Treatment of Eumycetoma Patients with *S. aureus* Co-infection Does Not Affect the Hepatic Function

Ketoconazole administration in high doses is hepatotoxic and therefore patients require close monitoring of their hepatic functions during treatment [8]. Adding other medication to the ketoconazole treatment regime could further influence the hepatic functions. The total protein, albumin, globulin, bilirubin and the liver enzymes; alkaline phosphates, serum glutamic oxaloacetic transaminase (SGOT) and serum glutamate-pyruvate transaminase (SGPT) were measured at the start of the study, after one month, 3 months, 6 months and 12 months in this study. The addition of amoxicillin-clavulanic acid or ciprofloxacin to ketoconazole did not result in hepatic toxicity. This suggests the safety of this combination.

## Discussion

Previous studies and clinical observations documented that, bacterial co-infection is common in eumycetoma patients and *S. aureus* is the most commonly isolated bacterial species [5]. The bacterial infection may disturb the local environment, reduce drug efficacy, and could produce local metabolites that may induce changes affecting the healing process and increase drug resistance [19], [20]. Hence, it can be hypothesized that secondary bacterial infections can influence the therapeutic outcome of the antifungal treatment for eumycetoma patients.

In this study, *S. aureus* strains were tested for their susceptibility to a large group of antibiotics and amoxicillin-clavulanic acid and ciprofloxacin appeared to be the most effective ones. It is interesting to note that, amoxicillin-clavulanic acid eradicated the infection in most of the patients whereas the bacterial co-infection persisted in all patients in the ciprofloxacin group. The explanation of this is unclear but cases of ciprofloxacin resistant staphylococci were reported even during the drug investigational stage, and since its introduction into routine clinical use, there has been increasing reports of ciprofloxacin-resistant staphylococci [22]–[27]. It appears that once resistance developed, person-to-person transmission within an environment favorable to spread resulted in widespread dissemination of resistant strains [24], [27].

Amoxicillin-clavulanic acid treatment resulted in eradication of *S. aureus* co-infection in the affected patients, it is therefore not surprisingly, that clinical improvement in these patients manifested by reduction of lesion size, number of active sinuses and less disability compared to the ciprofloxacin and the ketoconazole monotherapy groups.

In untreated mycetoma, particularly those with lesions containing multiple active sinuses, disease progression results in further tissue damage and destruction. It is highly likely that the bacterial co-infection has a major role to play in the disease progression [28].

It can be extrapolated from this observation that, the improved clinical outcome is due to co-infection eradication leading to less disease progression in the amoxicillin-clavulanic acid group compared to the other groups.

In mycetoma, the criteria of clinical improvement include lesion's size reduction, reduced active sinuses, normal skin and reduced disability. All these were documented in amoxicillin-clavulanic acid group which is possibly due to eradiation of the co-infection. It is interesting to note that the clinical improvement was observed within the first three months of amoxicillin-clavulanic acid treatment. This was not observed in the ciprofloxacin group and it is likely to be due to the persistence of the secondary bacterial infection and its local effects on the lesions.

The results obtained in this study are encouraging as eradication of the co-infection was associated with better clinical outcome. However ketoconazole the gold standard treatment for mycetoma has a narrow safety margins. Van de Sande and associates [29], Van Belkum and colleagues [30], Kloezen and associates [31] showed in *in vitro* susceptibility testing, encouraging results with the new generation of the azole group. [32]–[35]. Further studies combining amoxicillin-clavulanic acid with voriconazole or posaconazole are recommended. Both voriconazole and posaconazole have a better safety-profile than the currently used ketoconazole [32]-[35] and therefore avoiding liver toxicity and hepatitis [36].

In conclusion, amoxicillin-clavulanic acid proved to be highly effective in eradication of *S. aureus* co-infection in patients with eumycetoma due to *M. mycetomatis* and was associated with good clinical outcome and safety profile with no reported hepatic toxicity. Combination of amoxicillin-clavulanic acid and new less toxic azole drugs is recommended to avoid the toxic effect of ketoconazole which was recently banned by the Food and Drugs Administration (USA) (http://www.fda.gov/drugs/drugsafety/ucm362415.htm).
